# Existence and possible roles of independent non-CpG methylation in the mammalian brain

**DOI:** 10.1093/dnares/dsaa020

**Published:** 2020-09-24

**Authors:** Jong-Hun Lee, Yutaka Saito, Sung-Joon Park, Kenta Nakai

**Affiliations:** Human Genome Center, The Institute of Medical Science, The University of Tokyo, Tokyo, Japan; Artificial Intelligence Research Center, National Institute of Advanced Industrial Science and Technology (AIST), Tokyo, Japan; AIST-Waseda University Computational Bio Big-Data Open Innovation Laboratory (CBBD-OIL), Tokyo, Japan; Department of Computational Biology and Medical Sciences, Graduate School of Frontier Sciences, The University of Tokyo, Kashiwa, Japan; Human Genome Center, The Institute of Medical Science, The University of Tokyo, Tokyo, Japan; Human Genome Center, The Institute of Medical Science, The University of Tokyo, Tokyo, Japan; Department of Computational Biology and Medical Sciences, Graduate School of Frontier Sciences, The University of Tokyo, Kashiwa, Japan

**Keywords:** Non-CpG methylation, hidden Markov model, neuro-epigenetics

## Abstract

Methylated non-CpGs (mCpHs) in mammalian cells yield weak enrichment signals and colocalize with methylated CpGs (mCpGs), thus have been considered byproducts of hyperactive methyltransferases. However, mCpHs are cell type-specific and associated with epigenetic regulation, although their dependency on mCpGs remains to be elucidated. In this study, we demonstrated that mCpHs colocalize with mCpGs in pluripotent stem cells, but not in brain cells. In addition, profiling genome-wide methylation patterns using a hidden Markov model revealed abundant genomic regions in which CpGs and CpHs are differentially methylated in brain. These regions were frequently located in putative enhancers, and mCpHs within the enhancers increased in correlation with brain age. The enhancers with hypermethylated CpHs were associated with genes functionally enriched in immune responses, and some of the genes were related to neuroinflammation and degeneration. This study provides insight into the roles of non-CpG methylation as an epigenetic code in the mammalian brain genome.

## 1. Introduction

For decades, DNA methylation was thought to occur only at CpG sites in mammalian cells. However, recent studies using advanced genome-wide sequencing show that non-CpG sites are also methylated in several types of mammalian cells.^1–3^ Methylated non-CpGs (mCpHs; H indicates A, C, or T), which are highly cell type specific^1^^,^^2^ and associated with the transcription of nearby genes,^3^ have emerged as important epigenetic markers.

The mCpHs have been studied widely in pluripotent stem cells (PSCs) and brain cells^4–7^; they are more abundant in these cells than in other cell types^8^ and exhibit unique enrichment patterns, including colocalization with histone modifications,^9^ depletion in regulatory regions,^10^ and accumulation in gene-body regions and transposons.^5^ These patterns are highly distinct between the PSCs and brain and are potentially linked to cell type-specific functions. For example, the CAG motif tends to be methylated in PSCs, whereas the CAC motif is preferentially methylated in brain tissues.^8^ Methylated CACs in brain tissues are the binding target of MECP2, the mutation of which causes a neurological disorder, Rett syndrome.^11^ In addition, mCpH enrichment in PSCs is positively correlated with cell differentiation capacity.^12^

The mCpHs are often positioned near methylated CpGs (mCpGs), and they show lower methylation levels.^4^ Because they colocalize with mCpGs, whether mCpHs alone affect cellular processes remains unclear. In mammalian cells, CpHs are methylated by de novo DNA methyltransferases DNMT3a and DNMT3b, which methylate CpGs preferentially.^7^^,^^13^ Because these enzymes are highly expressed in PSCs and brain cells,^8^ mCpHs are considered byproducts of enzyme hyperactivity.^7^^,^^14^^,^^15^

In this study, we investigated the functional relevance of mCpHs in PSCs and brain cells. To this end, we conducted a large-scale analysis using publicly available datasets encompassing 26 whole-genome bisulfite sequencing (WGBS) samples and 368 microarray samples, including DNMT-knockout samples, to elucidate the functional involvement of the enzymes. In addition, we developed a hidden Markov model (HMM) to systematically detect genomic regions in which CpG and CpH are differentially methylated, providing an opportunity to infer the functional importance of non-CpG methylation.

## 2. Materials and methods

### 2.1. Analysis of WGBS samples

WGBS data from human PSCs, human tissue cells (brain, lung, and spleen), and mouse ESCs (mESCs) were downloaded from Gene Expression Omnibus (GEO). We found 15 datasets of 5 independent studies that assayed postnatal non-neuro-disordered brains. Among them, 14 datasets of 4 studies provided bisulfite-non conversion rates. We collected 9 datasets of 3 studies^1–3^ in which more than 50% of cytosines were detected (Supplementary Table S1). To our knowledge, this preparation of 9 datasets is the best effort to conduct analyses at this study with the available relevant WGBS samples in GEO (as of October 2016).

After filtering out low-quality reads using the FASTX-toolkit (http://hannonlab.cshl.edu/fastx_toolkit/) and duplicate reads using Samtools,^16^ the reads were mapped to the reference genomes (hg19 and mm10) using three bisulfite-read mappers: Bismark, ^17^ BSMAP, ^18^ and BS-seeker2.^19^ Then, cytosine bases in which more than five reads were aligned by at least two mappers were identified.^20^

### 2.2. Identification of methylated cytosine

We identified methylated cytosines from the CpG and CpH contexts using different thresholds because their average methylation levels are significantly different.^4^^,^^5^ In the CpG context, the methylation level ${Me}_{i}$ at the *i*-th cytosine base was calculated as follows:

$${Me}_{i}=\frac{\sum_{j}m_{i,j}}{\sum_{j}n_{i,j}}-R,$$

where *j* stands for one of the three mappers, *R* is the non-conversion rate based on the spiked-in of fully unmethylated DNA of lambda phage (Supplementary Table S1), and *m* and *n* represent the number of methylated (i.e. unconverted) reads and the total number of mapped reads, respectively. The CpGs were defined as methylated if *Me* > 0.8. For DNMT1-, DNMT3a-, and DNMT3b-reintroduced mESCs; however, CpGs were defined as methylated if *Me* > 0.5 because of the generally decreased methylation level. In the CpH context, the *i*-th cytosine base was defined as methylated by the significance of the binomial test, which was defined as follows:

Prmini,R=nimiRmi(1-R)ni-mi,

where *m* and *n* represent the number of methylated reads and the total number of mapped reads, respectively, and *R* stands for the non-conversion rate. Then, methylated reads were randomly generated from a binomial distribution given by *R* and $n_{i}$ (null data), and the false discovery rate (FDR) $\left(i.e.\;\;\frac{\mathrm{mCs}\;in\;\mathrm{null}\;\mathrm{data}}{\mathrm{mCs}\;in\;\mathrm{real}\;\mathrm{data}}\right)$ was calculated for the mC sets defined by different *P*-values (1.0e-2–1.0e-6). Finally, the *P*-value threshold was set as 1.0e-5, which allows an FDR < 0.01 in all samples. The mCpG level in a genomic region is defined as average *Me* at CpGs in the region, and mCpH level is defined as number of mCpHs divided by number of CpHs in the region. The X and Y chromosomes were removed from the methylome to eliminate potential bias derived from gender.

### 2.3. Analysis of DNA methylation microarray data

The methylation profiles generated from Illumina HumanMethylation450 BeadChip were analyzed to reproduce the differential mCpH distribution between the human brain and PSCs. We downloaded 177 brain samples from BrainSpan (http://www.brainspan.org/) and 191 PSC samples from GSE59091.^21^ Among 3,091 CpH sites in the arrays, 1,079 sites had no CpGs within 100 bp (CpG-distal), and an average of 26 sites had >1 mCpG (mCpG-proximal).

### 2.4. Designing an HMM

An empirical HMM was designed to detect the differentially methylated regions (DMRs) of CpG and CpH (CpG-CpH DMRs) in each human sample. Specifically, the whole genome was segmented into 180-bp-long bins and the emission probability *E* for a state *j* at the *i*-th bin was calculated as follows:

$E_{i,j}=\mathrm{Pr}\left(m_{i}|n_{i},\theta_{i,j}\right)\;∼\;\mathrm{Bin}\left(m_{i}|n_{i},\theta_{i,j}\right)$,

where $m_{i}$ and $n_{i}$ are the number of methylated reads and the total reads aligned to the cytosine at *i*-th bin, respectively. The state *j* consists of P, N, and U, representing the correlation state of methylation levels at each bin: P, positive correlation between CpH and mCpG methylation; N, negative correlation between CpH and CpG methylation; and U, uncorrelated.

The probabilities of the three states were differentiated by optimizing θ_i, j_ (${\hat{ɵ}}_{i,j}$). In the U-state, the CpH methylation is not correlated to the CpG methylation; thus, ${\hat{ɵ}}_{i,U}$ is the ratio of mCpH reads over total reads at CpHs in the whole-genome *w* as follows:

$${\hat{ɵ}}_{i,U}={\hat{ɵ}}_{w}=\frac{\sum_{i}m_{i}}{\sum_{i}n_{i}}.$$

Then, ${\hat{ɵ}}_{i,j}$ for the P and N states, *j* = {P, N}, is estimated by maximizing the posteriori estimation, where a prior probability *Pr* is modeled as a beta distribution as follows:

$$\begin{array}{c} {\hat{\theta }}_{i,j}=\mathrm{argma}x_{\theta_{i,j}}Pr\left(\theta_{i,j}|m_{i},n_{i}\right)\propto \mathrm{argma}x_{\theta_{i,j}}Pr\left(m_{i},n_{i}{|\theta }_{i,j}\right)Pr(\theta_{i,j})\\ =\mathrm{argma}x_{\theta_{i,j}}\theta_{i,j}{}^{\left(m_{i}+\alpha_{i,j}\right)}{\left(1-\theta_{i,j}\right)}^{\left(n_{i}-m_{i}+\beta_{i,j}\right)}\\ =\frac{m_{i}+\alpha_{i,j}}{ni+\alpha_{i,j}+\beta_{i,j}} \end{array}$$

Here, *α* and *β* are used as pseudo-counts to reflect the CpG methylation status at each bin. *α* and *β* are regulated by adjusting the mean *M* of the distribution at the *i*-th bin as follows:

$$\begin{array}{c} M_{i,P}={\hat{\theta }}_{U}+{\;\text{SD}}_{mH}\times \frac{mG_{i}-MED_{mG}}{{\text{SD}}_{mG}},\\ M_{i,N}={\hat{\theta }}_{U}-{\;\text{SD}}_{mH}\times \frac{mG_{i}-MED_{mG}}{{\text{SD}}_{mG}}, \end{array}$$

where ${mG}_{i}$ is the mCpG reads over all reads at CpGs in the *i*-th bin. ${\mathrm{SD}}_{mG}$ and ${\mathrm{MED}}_{mG}$ are the standard deviation and median of the mGs in the whole genome, respectively. ${\mathrm{SD}}_{mH}$ is the standard deviation of the mCpH reads over all reads at CpHs in each bin among whole genome. In brief, if the CpG methylation level at the *i*-th bin is higher than the genome-wide average, $M_{i,A}$ is higher than ${\hat{ɵ}}_{U}$, whereas $M_{i,B}$ is lower than ${\hat{ɵ}}_{U}$. Because $M=\frac{\alpha }{\alpha +\beta }$, we defined *α* and *β* as follows:

$$\begin{array}{c} \alpha_{i,j}=n_{i}\times M_{i,j},\\ \beta_{i,j}=n_{i}-\alpha_{i,j}, \end{array}$$

where *n_i_* is the number of reads at all CpGs in the *i*-th bin.

The *E* for bins in which the number of reads aligned at CpGs and CpHs was >10 was calculated. In addition, to ensure the continuity of the Markov model, the genomic region was divided if the continuous undetected bins were longer than 100,000 bp, and the HMM was applied separately. The probability of state transition was estimated using an expectation-maximization (EM) algorithm that repeats the EM steps until the difference between the previous and current transition probabilities of all state transactions is <5e-4.^22^ Then, the Viterbi algorithm that finds an optimal path among the states was applied, and the bins were re-defined to P-, N-, or U-state. The consecutive bins were linked if they were in the same state and the distance between them was <3 bins (540 bp). Finally, the N-state regions were defined as CpG–CpH DMRs.

### 2.5. Preparation of a simulated dataset

To validate the performance of the HMM, we generated an artificially methylated human chromosome 19 with randomly generated N states (equivalent to CpG-CpH DMRs) and compared the precision and recall rate of the N state defined by emission probability only and by transition probability learning and Viterbi decoding. Specifically, pseudo-bisulfite–treated reads were uniformly distributed with the average read depth of 10 to the human chromosome 19. Then, the read numbers at cytosine bases of each 180 bp bin were added. For the *i*-th bin, the number of unmethylated reads *un_m* at the CpG or CpH site *j* was randomly chosen from a binomial distribution provided by the total read number *n* and a modeled methylation level *Me* as follows:

$$\mathrm{Bin}\left({un\_m}_{i,j}|n_{i,j},{Me}_{i,j}\right),$$

where *Me_i_*_,CpG_ is a uniform random number distributed between 0 and 1. The *Me_i,_*_CpH_ in P-state is *Me_CpG_* × 0.1, and that in N state is (1 − *Me_CpG_*) × 0.1, reflecting the lower methylation level at CpHs relative to CpGs.

We generated 500 artificial sets containing 10–10,000 randomly distributed N-state regions, the length of which varied randomly from 1 to 100 bins. Then, whether the HMM could properly detect the N state by precision and recall rates was determined as follows:

$$\begin{array}{c} P\mathrm{recision}=\frac{TP}{TP+FP}\\ R\mathrm{ecall}=\frac{TP}{TP+FN}, \end{array}$$

where TP is the true-positive, FP the false-positive, and FN the false-negative rates of the detected N-state region.

### 2.6. Preparation of genome annotation

The annotation for hg19 was downloaded from a previous study^23^ including Enhancer (FANTOM5 robust enhancer),^24^ SuperEnhancer,^25^ WeakEnhancer CTCF,^26^ and Conserved.^27^ The promoter (±5,000 bp from the transcription start site), 5′-untranslated (5′-UTR), 3′-UTR, coding, CpG island (CGI), and CGI shore (±200 bp from CGI) regions were downloaded from the UCSC genome browser (https://genome.ucsc.edu/). The enhancers were obtained from a broad range of cell types and were not specific to brain tissues.

### 2.7. Identification of hyper- and hypo-mCpH DMRs

The mCpH level for each DMR was calculated, and DMRs in which CpHs were hyper- or hypo-methylated were identified using the following criteria: >2 fold-change (FC) relative to the mCpH level in the whole genome (hyper-mCpH-DMR), and <0.5 FC relative to the mCpH level in the whole genome (hypo-mCpH-DMR). The overlap between the DMRs and the annotated genomic regions was extracted using Bedtools intersect.^28^

### 2.8. Analysis of transcription factor binding sites

The transcription factors (TFs) binding to the hyper- and hypo-mCpH DMRs overlapping with transcription regulatory regions (i.e. promoter and enhancer) were analyzed. First, genomic regions in which enhancers and promoters overlapped with the hyper- or hypo-mCpH-DMRs in each sample were extracted. Then, the regions overlapped in at least two brain samples were selected for further analysis. Then the enriched TF binding sites in the regions common in at least two samples were analyzed using Homer^29^; the peak size was set at 200 bp (default) and repeated regions were filtered out. The input sequence was extracted from the transcribed strand for the promoter regions and from both strands for the enhancer regions. In addition, the sequences in all promoter and enhancer regions were used as background sequence. Significantly enriched binding motifs of known TFs were extracted from knownResults.txt with a Benjamini-Hochberg FDR < 0.01.

### 2.9. Analysis of gene ontology and pathway enrichment

Enrichment of gene ontology (GO) terms (Biological Process, Molecular Function, and Cellular Component) and pathways [Kyoto Encyclopedia of Genes and Genomes (KEGG)] among genes targeted by the hyper- and hypo-mCpH-promoters and enhancers was analyzed. First, common hyper-/hypo-mCpH promoters and enhancers in 2–8 brain samples were extracted. Then, gene sets regulated by the promoter sets (derived from the different number of brain samples) were extracted from the RefSeq database (https://www.ncbi.nlm.nih.gov/refseq/) and gene sets regulated by the enhancer sets were extracted from Fantom5 human enhancer track (http://slidebase.binf.ku.dk/human_enhancers/presets). Of note, the linkage between genes and enhancers was derived from their co-expression in multiple cell and tissue types captured by CAGE-seq.^24^ In addition, the gene sets consisting of >3,000 genes were removed. Then, the enriched GO terms and KEGG pathways were analyzed using DAVID^30^; the background gene set was all genes in hg19, and the terms with Benjamini–Hochberg FDR < 0.01 were selected as significantly enriched.

## 3. Results

### 3.1. Differential distribution of mCpGs and mCpHs in PSCs and brain cells

To capture the genome-wide methylation status at each CpG and CpH site, human WGBS datasets were prepared from PSCs and brain cells, which contain abundant mCpHs, as well as from lung and spleen cells as controls (Supplementary Table S1). WGBS reads were analyzed using our statistical method, which integrates the outcomes from three tools; Bismark, BSMAP, and BS-seeker2. Methylation levels were quantified for up to 73% of CpGs and up to 96% of CpHs throughout the genome (Supplementary Table S1).

**Figure 1:**
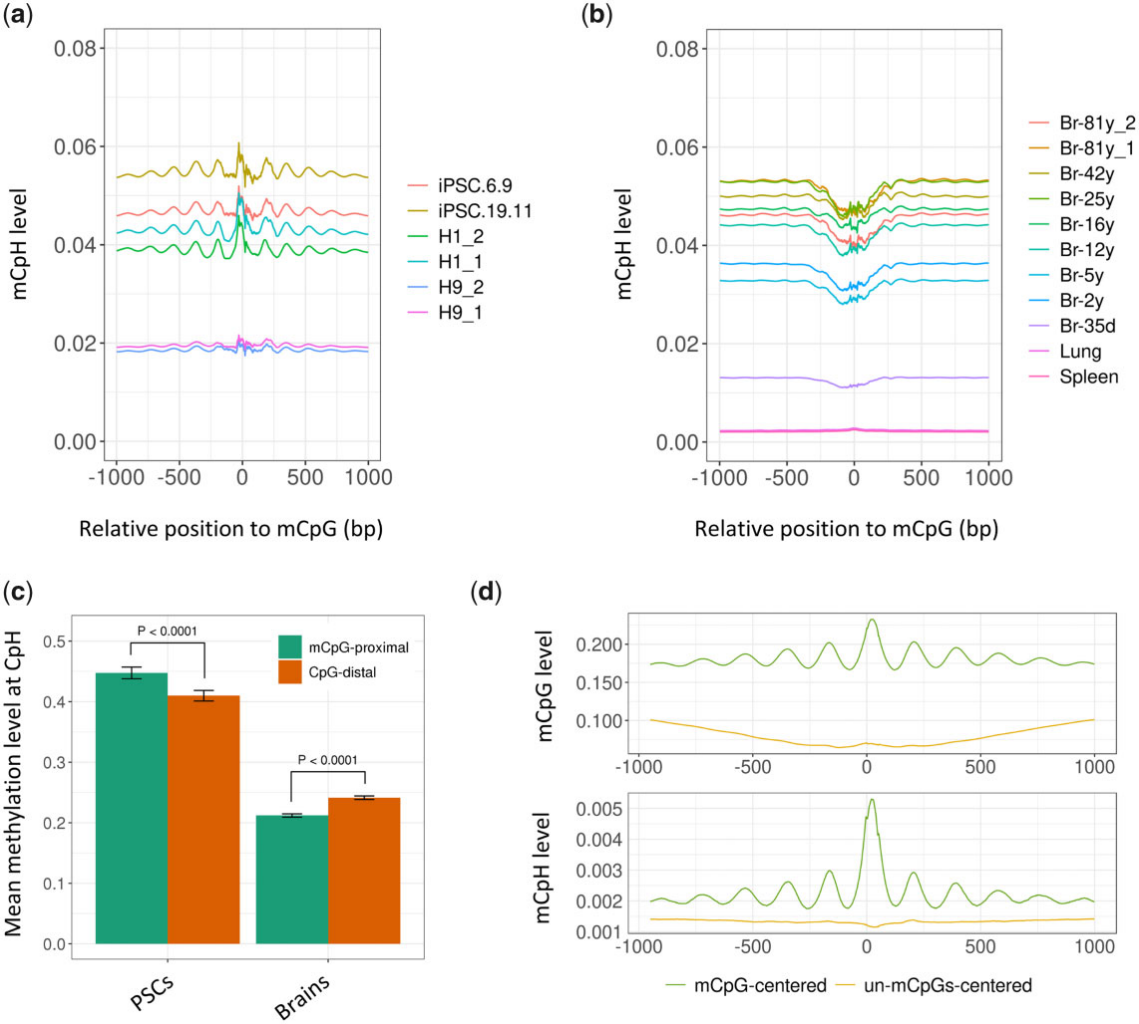
(a) Distribution of mCpH levels around mCpGs in human PSCs. (b) Distribution of mCpH levels around mCpGs in brain and control tissues; in the cell identifiers, the numbers following an underscore indicate biological replicates. (c) Difference in mCpH levels at mCpG-proximal (<±100 bp) and CpG-distal (>±100 bp) CpH sites in 177 brain tissues and 191 PSCs; the *P* value was calculated by the Wilcoxon signed-rank test. (d) Distribution of methylation levels around mCpGs and unmethylated CpGs (methylation level <0.05) in DNMT1-KO mESCs. For (a), (b), and (d), the center (un) mCpG is derived from both DNA strands, and the methylation level at CpHs (or CpGs) in the same strand of the mCpG is averaged by 50-bp window sliding. The x-coordinate follows the 5′ to 3′ direction, and the relative position represents flanking 1K bp for each indicated cytosine in position zero, as used in other studies.^4^^,^^5^^,^^8^ Br, brain.

The positional correlation between mCpHs and mCpGs was investigated. As shown in Fig. 1a and b, CpHs in PSCs were hypermethylated near mCpGs, whereas those in brain cells were hypomethylated near mCpGs. Because the position of mCpGs in the two cell types may determine the distinctive distribution of mCpHs, we focused on mCpGs present in both PSCs and brain cells and obtained consistent results (Supplementary Fig. S1a and b). In addition, a different mCpH pattern was observed at the CAC and CAG motifs (Supplementary Fig. S1c–e), which showed preferential methylation in PSCs and brain tissue, respectively.^11^. This suggests that the observed pattern was not caused by a positional difference of the preferentially methylated CpHs in the two cell types. To confirm the differential distribution of mCpHs, we analyzed 368 microarray-based DNA methylation datasets and compared the mCpH levels in mCpG-proximal sites (within 100 bp of mCpG) with the mCpH levels in CpG-distal sites (>100 bp away from CpG). The average CpH methylation level from the microarray data was higher than that from the sequencing data, as reported in a previous study,^10^ because the array includes CpHs identified as preferentially methylated in human stem cells. As shown in Fig. 1c, mCpG-proximal mCpH levels were significantly higher than CpG-distal mCpH levels in PSCs, but not in brain cells. These results indicate that mCpHs colocalize with mCpGs in PSCs, but not in brain tissue.

In addition, a 180 bp periodicity of mCpH levels was observed in PSCs (Fig. 1a, Supplementary Fig. S1) and brain cells (Supplementary Fig. S1). This may reflect the nucleosome positioning that regulates the accessibility to DNMTs^31^ and indicates that methyltransferases move along the DNA and methylate CpGs and CpHs simultaneously, as reported previously.^32^^,^^33^

### 3.2. Involvement of DNMT activities in the methylation processes

CpH methylation is catalyzed by DNMT3a and DNMT3b^7^^,^^13^^,^^34^, which are differentially expressed: DNMT3a is highly expressed in brain cells, whereas DNMT3b is preferentially expressed in PSCs.^8^ Therefore, we hypothesized that the mCpH distribution pattern (Fig. 1a and b) may be determined by the differential activities of DNMT3a and DNMT3b. To test this hypothesis, we first analyzed WGBS data from Dnmt1-KO (knockout) mESCs, in which DNA methylation is primarily catalyzed by Dnmt3a and Dnmt3b. As shown in Supplementary Table S1, deletion of Dnmt1 substantially decreased mCpG levels, but not mCpH levels, which is consistent with previous studies reporting the maintenance and de novo CpG methylation by Dnmt1.^13^ In addition, the co-localization of mCpH with mCpG was more pronounced, and mCpH levels were higher near mCpGs and decreased with increasing distance, showing clear 180 bp periodicity (Fig. 1d). This result indicates that Dnmt3a and Dnmt3b methylate CpGs and CpHs simultaneously.

Next, to evaluate methylation by DNMT3a and DNMT3b separately, we used mESCs in which either Dnmt3a or Dnmt3b was re-introduced after knocking out Dnmt1, Dnmt3a, and Dnmt3b.^31^ Despite the low quality of the data obtained (detected number of CpGs and CpHs in Supplementary Table S1), higher mCpH levels were observed near mCpGs in both Dnmt3a- and Dnmt3b-re-introduced samples (Supplementary Fig. S2a), indicating that each of these enzymes mediates the co-localization of mCpGs and mCpHs. In addition, mCpGs and mCpHs were positively correlated in both DNMT3a- and DNMT3b-KO hESCs (Pearson correlation coefficient = 0.38 and 0.32, respectively; Supplementary Fig. S2b).

These results demonstrate that DNMT3a and DNMT3b can singly mediate the colocalization of mCpGs and mCpHs. This suggests that the depletion of mCpHs near mCpGs in brain cells is not caused by DNMT3a hyperactivity but rather is the result of the complex epigenetic mechanisms in the mammalian brain.

### 3.3. Detection of differentially methylated CpGs and CpHs

Because both DNMT3a and DNMT3b generated a positive correlation between mCpH and mCpG levels at the genome-wide scale, we assumed that the negative correlation between mCpH and mCpG levels in brain tissues is highly locus specific. Such genomic regions were designated as CpG–CpH DMRs. To detect CpG–CpH DMRs, several issues must be considered. First, because average methylation levels differ dramatically between CpGs and CpHs, the methylation states at CpGs and CpHs must be evaluated in different scopes. Second, because average methylation levels also vary among cell types (Fig. 1a and b), the methylation criteria need to be adjusted for each cell type. Finally, the methylation data show positional continuity with the 180 bp periodicity, which is useful for detecting DMRs.

**Figure 2:**
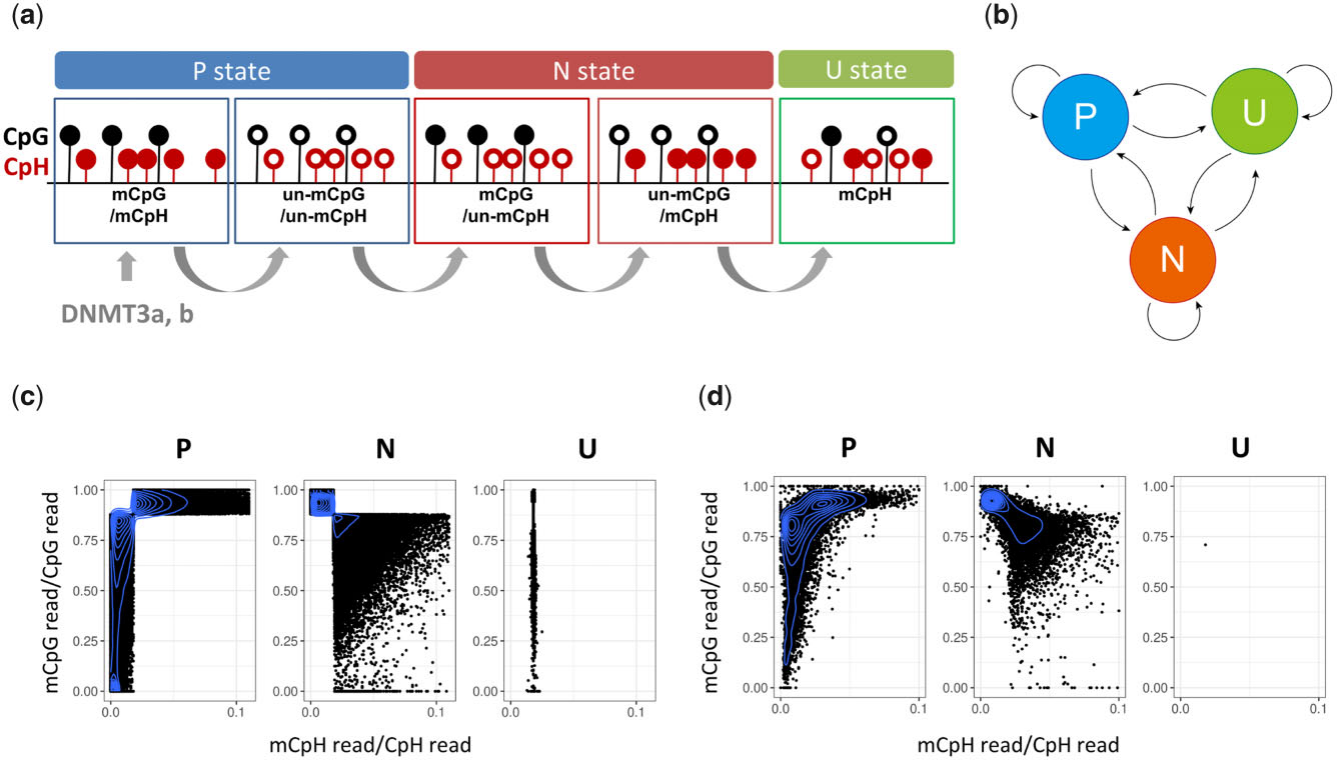
(a) Schematic representation of the three states in HMM. In the P state, both CpGs and CpHs are methylated or unmethylated, resulting in a positive correlation between the mCpG and mCpH levels. In the N state, either CpGs or CpHs are methylated, resulting in a negative correlation between the levels. In the U state, the levels are not correlated. The bin size was set to 180 bp because DNMT3a and DNMT3b methylate DNA strands with a ∼180 bp periodicity. The N-state bins are considered CpG–CpH differentially methylated regions (CpG–CpH DMRs). (b) Schematic diagram of the state transition in the HMM. The transition probability between the states was estimated using the EM algorithm. (c) Distribution of the mC reads/total C reads at CpG sites (*y*-axis) and CpH sites (*x*-axis) aligned on each bin. The states are designated by the highest emission probability. (d) Distribution of mC reads/total C reads at CpG sites (*y*-axis) and CpH sites (*x*-axis) aligned on the P-, N-, and I-state regions designated by the Viterbi algorithm. For visualization, (c) and (d) were drawn using chromosome 19 of sample Br-81y_1.

To address these issues, we adopted an HMM approach in which the whole genome was segmented into 180 bp bins, and each bin was categorized into one of three states: P, N, and U (Fig. 2a). In P-state regions, mCpH and mCpG levels correlated positively (P). In N-state regions, the levels correlated negatively (N), namely, they were equivalent to CpG–CpH DMRs. In U-state regions, the levels were uncorrelated (U); therefore, the mCpH level remained at the average level and was not affected by the surrounding mCpG level.

We calculated the probability that a bin belongs to any of the three states (emission probability) and the probability of state transition modeling the positional continuity between two neighboring bins (Fig. 2b). To represent variation of mCpG and mCpH patterns among cell types, the transition probability was trained by the EM algorithm for each cell type. After training, the HMM assigned the most probable state for each bin using the Viterbi algorithm (Supplementary Fig. S3a; see Supplementary Methods). As shown in Fig. 2, the state classification of bins using the highest emission probability (Fig. 2c) and the Viterbi algorithm (Fig. 2d) demonstrated that mCpG and mCpH levels were correlated positively in the P states and negatively in the N states. After Viterbi decoding, the U states were absorbed by the P or N states, as the probability of transition from U to U is lower (Supplementary Fig. S3b).

The HMM was designed to properly define the bin states by considering methylation information in and around the bin (Supplementary Fig. S3a). To confirm its performance, we generated an artificially methylated human chromosome 19 with randomly generated N states and compared precision and recall rates of the N state, defined by only emission probability or by the transition learning and Viterbi decoding (Supplementary Fig. S3c). Both precision and recall rates increased after Viterbi decoding, indicating that definition of CpG–CpH DMRs was more accurate using whole HMM workflows than using the emission probability alone.

### 3.4. Functional analysis of brain CpG–CpH DMRs

**Figure 3:**
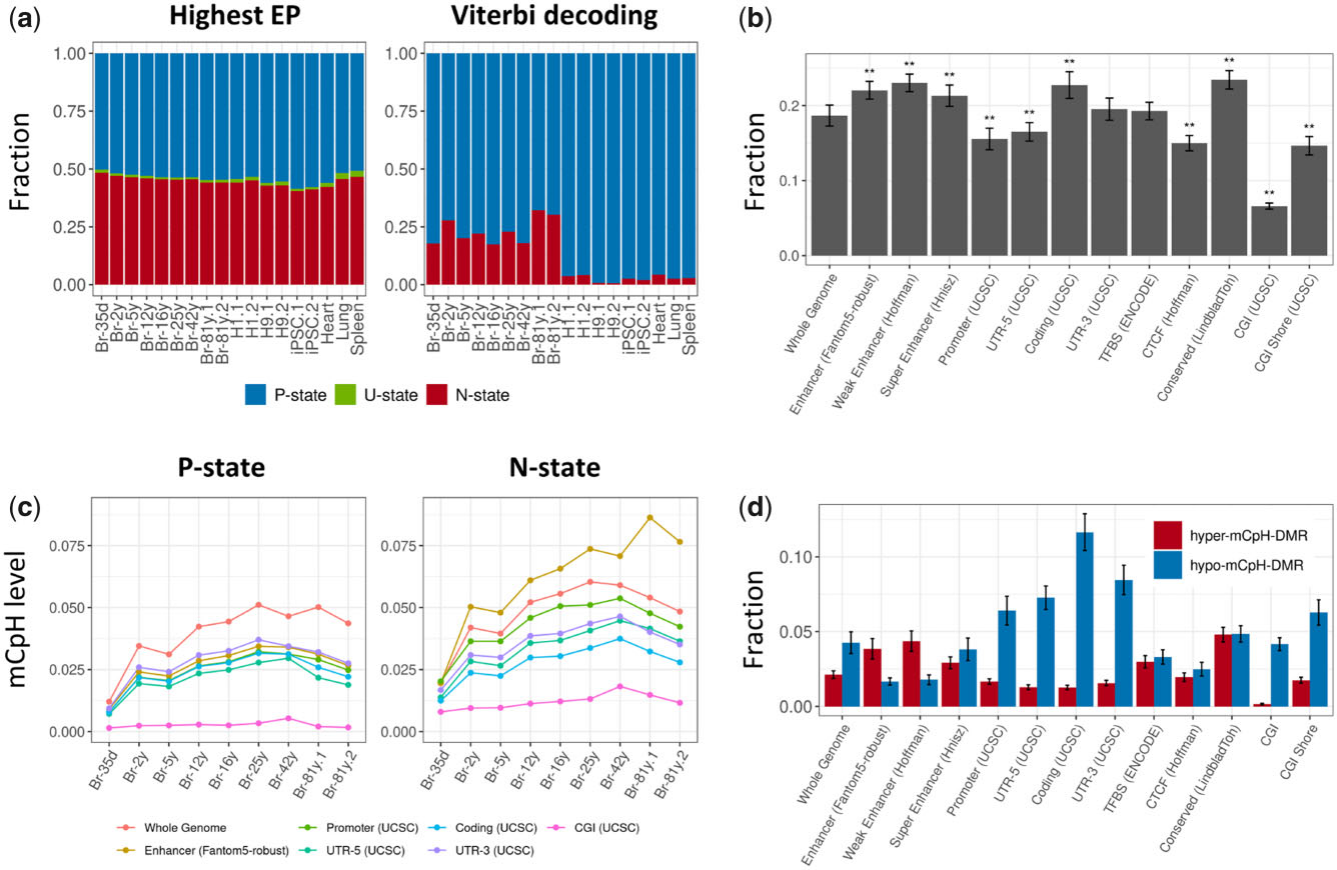
(a) Fractions of the P-, N-, and U-state regions detected by the highest emission probability (left) and the Viterbi algorithm (right). (b) Fractions of the whole-genome and of the annotated regions that are overlapped with DMRs in brain samples. (c) Distribution of mCpH levels in P- and N-state regions that are overlapped with the annotated regions in brain samples. (d) Fractions of the whole-genome and of the annotated regions that are overlapped with hyper- and hypo-mCpH-DMRs. The bars indicate standard error. The *P* values obtained with the Student’s *t*-test are denoted as *P < 0.01, **P < 0.001. EP, emission probability.

The HMM was applied to each of the WGBS samples, and the states of all genomic regions were estimated. Approximately 20% of the genomic bins in brain samples were defined as CpG–CpH DMRs, which was a high proportion compared with that in other cell types (Fig. 3a). Moreover, the DMRs were relatively conserved among brain samples but differed from others (Supplementary Fig. S4a). In the DMRs of the brain samples, the CAC motif, followed by the other CA motifs and CTC motif, were mainly methylated, which is similar to the P-state region (Supplementary Fig. S4b).

The fraction of DMRs in a subset of known genomic elements was analyzed (Fig. 3b). The DMRs overlapped significantly with genomic regions conserved in mammals, coding regions, and enhancer regions, whereas they overlapped less with promoter, 5′-UTR, CTCF, CpG island, and CpG island shore regions. Remarkably, the enhancers marked by DMRs showed high mCpH levels, and the mCpH levels largely increased with age (Fig. 3c), indicating the potential role of mCpHs in the DMRs in brain aging.

In further analysis, we collected DMRs in which CpHs were hyper- or hypo-methylated (hyper-mCpH-DMR and hypo-mCpH-DMR, respectively). Hyper-mCpH-DMRs largely overlapped with enhancer regions, whereas hypo-mCpH-DMRs overlapped with promoter and coding regions (Fig. 3d), indicating distinct mCpH density between the DMRs in distal and proximal regulatory regions. The enrichment of TF binding motifs specific for the hyper-mCpH-DMR and hypo-mCpH-DMR overlapped with the enhancer and promoter regions (hyper-mCpH enhancer, hypo-mCpH enhancer, hyper-mCpH promoter, and hypo-mCpH promoter, respectively) was analyzed. The TF binding motifs were enriched in the hyper-mCpH enhancers and the hypo-mCpH promoters, rather than the hypo-mCpH enhancers or the hyper-mCpH promoters (Supplementary Fig. S5a and b).

**Figure 4:**
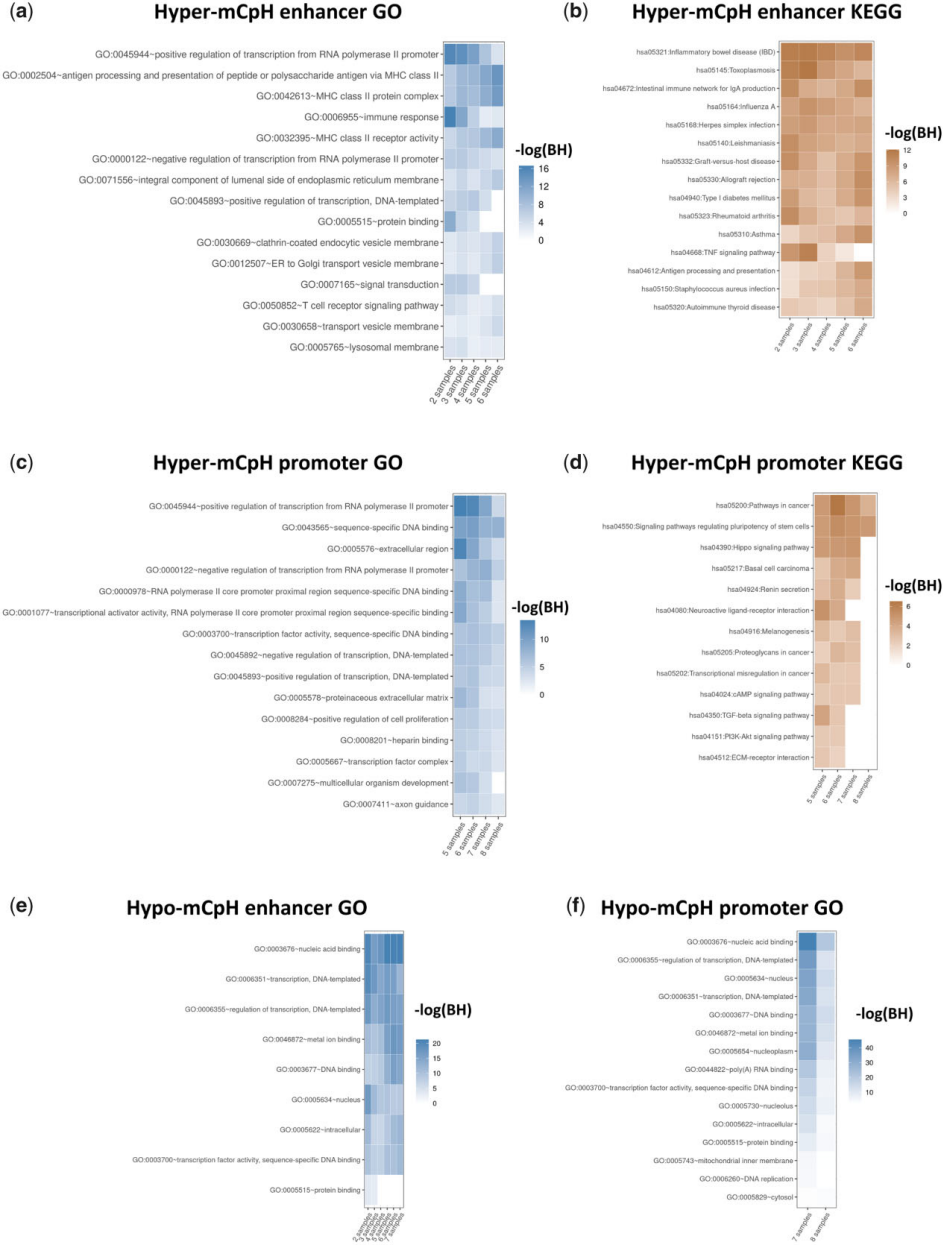
Results of GO and KEGG pathway enrichment analysis of genes regulated by the enhancers and promoters overlapped with hyper- and hypo-mCpH DMRs [hyper-mCpH enhancer (a and b), hyper-mCpH promoter (c and d), hypo-mCpH enhancer (e), and hypo-mCpH promoter (f)]. Because the hyper- and hypo-mCpH DMRs are distinct among brain samples, the promoters and enhancers overlapped with the DMRs in N samples and the corresponding gene sets (*x*-axis) were extracted. The GO terms and pathways (*y*-axis) are sorted by the sum of the -log(BH) from the gene sets, and the top 15 are shown. Only gene sets with at least one significant term or pathway are included. For example, there was no significant GO term for the enhancers overlapped with hyper-mCpH DMR in one, seven, and eight brains samples in common (a).

Next, we analyzed GO and KEGG pathway enrichment of the genes regulated by the enhancers and promoters. The hyper-mCpH enhancers were involved in transcription by RNA polymerase II (GO: 0045944), immune response (GO: 000955), and MHC class II protein complex (GO: 0042613, GO: 0032395, and GO: 0002504), activating the immune response in antigen-presenting cells^35^ (Fig. 4a). KEGG pathways enriched in the hyper-mCpH enhancers included immune-related diseases and pathways, such as inflammatory bowel disease (hsa05321), intestinal immune network for IgA production (hsa04672), and virus infections (hsa05164 and hsa05168) (Fig. 4b). The hyper-mCpH promoters were mostly involved in DNA binding and transcription activity, and weakly associated with axon guidance (GO: 0007411) (Fig. 4c). In addition, the enriched KEGG pathways included neuroactive ligand-receptor interaction (hsa04080), although the strongest signal corresponded to cancer-related pathways (Fig. 4d). The GO terms enriched in hypo-mCpH enhancers and hypo-mCpH promoters were mostly related to DNA binding and cellular housekeeping activities (Fig. 4e and f). There was no significant KEGG term enriched in both hypo-mCpH enhancers and promoters. The enhancers, promoters, corresponding genes, and DMR status in each brain sample are described in Supplementary Tables S3–S6.

**Figure 5:**
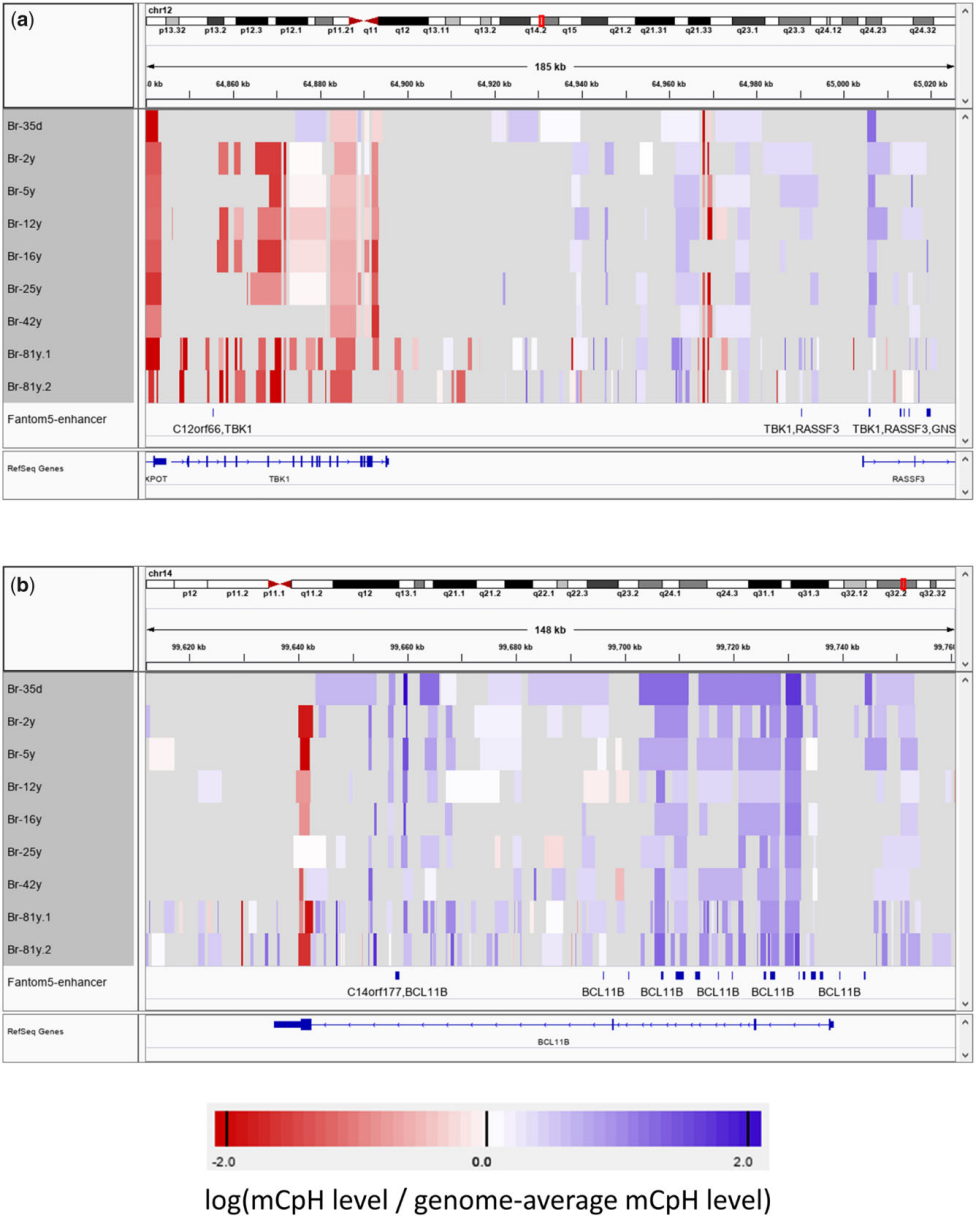
The Integrative Genomics Viewer capture shows examples of mCpH levels in the DMRs around TBK1 (a) and BCL11B (b) in brain samples. Color indicates log-FC of the mCpH level +0.0001 (to avoid log0) against the genome-wide average. The grey regions indicate P-state, U-state, or undetected bins.

Although there was no significantly enriched GO term or pathway among genes marked by the hyper-mCpH enhancer in more than six brain samples, some of the genes were associated with neurodegeneration via the regulation of virus infection (Supplementary Table S3). For example, TANK Binding Kinase 1 (TBK1) is a key gene regulating infection by multiple viruses, such as herpes simplex virus 1 (has 05168) and Epstein-Barr virus (hsa05169), and mutations in TBK1 activate the pathology of amyotrophic lateral sclerosis^36^ and early-onset Alzheimer’s disease.^37^ The putative enhancers of the gene were marked by hyper-mCpH DMR in seven of the eight brain samples (Fig. 5a, Supplementary Table S3). On the other hand, B-cell lymphoma/leukemia 11B (BCL11B) inhibits HIV transcription by recruiting a chromatin-modifying complex,^38^ and its disruption may cause Huntington’s disease and Alzheimer’s disease.^39^ The intronic enhancer regions of this gene were also marked by hyper-mCpH DMR in most of the brain samples (Supplementary Table S3, Fig. 5b).

Taken together, these observations suggest that the hypermethylation of CpHs at CpG–CpH DMRs is involved in immune responses and neuroactivity through the regulation of enhancer activity, and potentially associated with neurodegeneration.

## 4. Discussion

The mCpGs and mCpHs tend to colocalize in mammalian cells, casting doubt on the biological relevance of mCpHs.^7^^,^^14^^,^^15^ However, in this study, analysis of large-scale methylation data revealed that the patterns of these two methylation types are distinct in PSCs and brain cells. In particular, mCpHs were markedly depleted near mCpG sites in brain cells, but not in PSCs. Because our analysis of DNMT-KO samples indicated that this characteristic was not entirely attributed to the activity of DNMTs, we hypothesized that the co-methylation of CpHs with CpGs occurs in a cell type-specific manner and that these methylation processes have an unknown functional meaning.

To systematically detect the DMRs of CpGs and CpHs, we designed an HMM that was successfully applied in previous studies.^13^^,^^22^^,^^33^ The results showed that the DMRs are remarkably abundant in brain tissue compared with other tissue types (Fig. 3a), and relatively conserved among brain samples. The CpH methylation level in the DMR with the putative enhancer region increased with brain age (Fig. 3c), indicating its potential role in brain aging. A DMR is a genomic region in which either mCpH or mCpG levels are intensified. Thus, to confirm the mCpH depletion near mCpGs, we investigated the DMRs marked by hyper- and hypomethylation of CpHs. The hypo-mCpH DMRs occupied a larger portion of the whole genome than the hyper-mCpH DMRs, and were especially enriched in the promoter and coding regions (Fig. 3d). However, the hyper-mCpH DMRs considerably overlapped with enhancer regions, and the putative target genes of these enhancers are specifically involved in the immune response, the disruption of which in the brain increases the risk of neurodegenerative disorders.^40^

The present in-silico analyses provide evidence supporting the existence of mCpHs that are uncorrelated with mCpGs in the brain, as well as the potential functional association of mCpHs with neurodegeneration via the regulation of immune activity. However, the study had several limitations and issues that need to be solved. First, the mechanisms underlying the mutually exclusive methylation at CpGs and CpHs in the brain remain unclear. The mechanisms may involve complex DNA methylation and demethylation processes that orchestrate gene regulation, which are not completely understood.^34^^,^^41^ Especially, the involvement of demethylases in the formation of cell type-specific CpG-CpH methylation patterns should be addressed using additional data. Second, the corresponding transcriptome should be analyzed to investigate the effect of mCpHs on gene expression. Last, WGBS of brain samples at single-cell resolution may help elucidate the distribution and function of DMRs in detail. Thus, to better understand the roles of non-CpG methylation, future studies should incorporate genomic and epigenomic assays in various cell types along with comprehensive computational analyses. 

## Supplementary data

Supplementary data are available at http://DNARES online.

## Acknowledgments

Computational resources were provided by the SHIROKANE supercomputer system at the Human Genome Center of the Institute of Medical Science at the University of Tokyo.

## Funding 

This research was supported by Japan Society for the Promotion of Science (JSPS) KAKENHI (grant numbers: 16H01403 and 18H04710). Y.S. was supported by JSPS KAKENHI (grant numbers: 17H06410, 19K20409, 19K06502, and 19K06077). S.P. was supported by JSPS KAKENHI (grant number: 20K06606).

## Conflict of interest

None declared.

## Data availability

All data generated are included in this article and its supplementary information files. The Perl scripts for building HMM are available at Github (https://github.com/christiario/HMM).

## Supplementary Material

dsaa020_Supplementary_Data
