# Supplementary material for: Existence and possible roles of independent non-CpG methylation in the mammalian brain
Source: DNA Res. 2020 Sep 24;27(4):dsaa020. doi: 10.1093/dnares/dsaa020 (PMC7750974; doi:10.1093/dnares/dsaa020)
Supplement: dsaa020_Supplementary_Data [file dsaa020_supplementary_data.zip › Supplementary_Legends.pdf]

Supplementary data

Figures

Figure S1

(a, b) Distribution of mCpH levels around CpGs commonly methylated in both PSGs and brain tissues. (c, d) Distribution of methylation levels at C followed by AG (CAG). (e, f) Distribution of methylation levels at C followed by AC (CAC). mCpG, methylated CpG; mCpH, methylated non-CpG; PSC, pluripotent stem cell. The values are averaged by 50-bp window sliding.

Figure S2

(a) Distribution of mCpH levels around mCpGs in wild-type and DNMTs-KO mouse ESCs. DNMT-KO+3a, KO samples with re-introduced DNMT3a; DNMT-KO+3b, KO samples with re-introduced DNMT3b. The values are averaged by 50 bp-long window sliding. (b) Density plot showing the distribution of mCpG and mCpH levels in 1 kb genomic regions. Regions containing >10 CpGs and 10 CpHs were considered. As the density increases, the color gradually changes based on the color scheme grey (lowest density)–blue–red–yellow (highest density). WT, wild type; ESC, embryonic stem cell; KO, knockout; PCC, Pearson correlation coefficient.

Figure S3

(a) Example of N-state region detection with the Viterbi algorithm. (b) Transition probabilities in human samples. Probability learning was performed within a chromosome block in which undetected consecutive bins were not longer than 100,000 bp. The error bar indicates the standard error of the transition probabilities from all the chromosome blocks. (c) Distribution of precision and recall rates of the results obtained by highest emission probability (left) and by the Viterbi algorithm (right). TP, true positive; FP, false positive, FN, false negative; *n*, the total number of randomly generated N-state regions (length of the regions are randomly set); Highest EP, the highest emission probability.

Figure S4

(a) Tile plot describing the Jaccard index of DMRs in all the possible pairs of WGBS samples. The definition of Jaccard index is $|A \cap B|/|A \cup B|$, where A and B indicate DMRs in each sample. To calculate this, we formatted the DMRs to a bed file and used "bedtools jaccard". (b) The average CpH methylation level

(number of mC/number of C) at each tri-nucleotide motif in P- and N-states.

Figure S5

The tile plot describes the significant binding of each transcription factor (TF) to the hypo-/hyper-mCpH enhancer (a) and promoter (b) regions. The results from Homer-known motifs enriched with Benjamini-Hochberg (BH)-corrected p-value <0.01 in at least one of the regions are shown. For (b), the TFs with p-value <1e-100 were selected for visualization. The total TFs and corresponding BH-corrected p-values are shown in Table S2.

Tables

Table S1

Description of WGBS data used in this study.

Table S2

Known motif and consensus enrichment (BH-corrected p-value) in hyper/hypo-mCpH-DMRs overlapped with enhancer and promoter regions in at least two brain samples.

Tables S3–6

Putative enhancers and promoters overlapped with hyper-/hypo-mCpH DMRs in at least two brain samples. Columns with brain samples indicate whether the DMRs are overlapped (1) or not (0). The last column shows the sum of the overlapped samples.
