# Supplementary figures and images for: Existence and possible roles of independent non-CpG methylation in the mammalian brain
Source: DNA Res. 2020 Sep 24;27(4):dsaa020. doi: 10.1093/dnares/dsaa020 (PMC7750974; doi:10.1093/dnares/dsaa020)

**Figure S1**

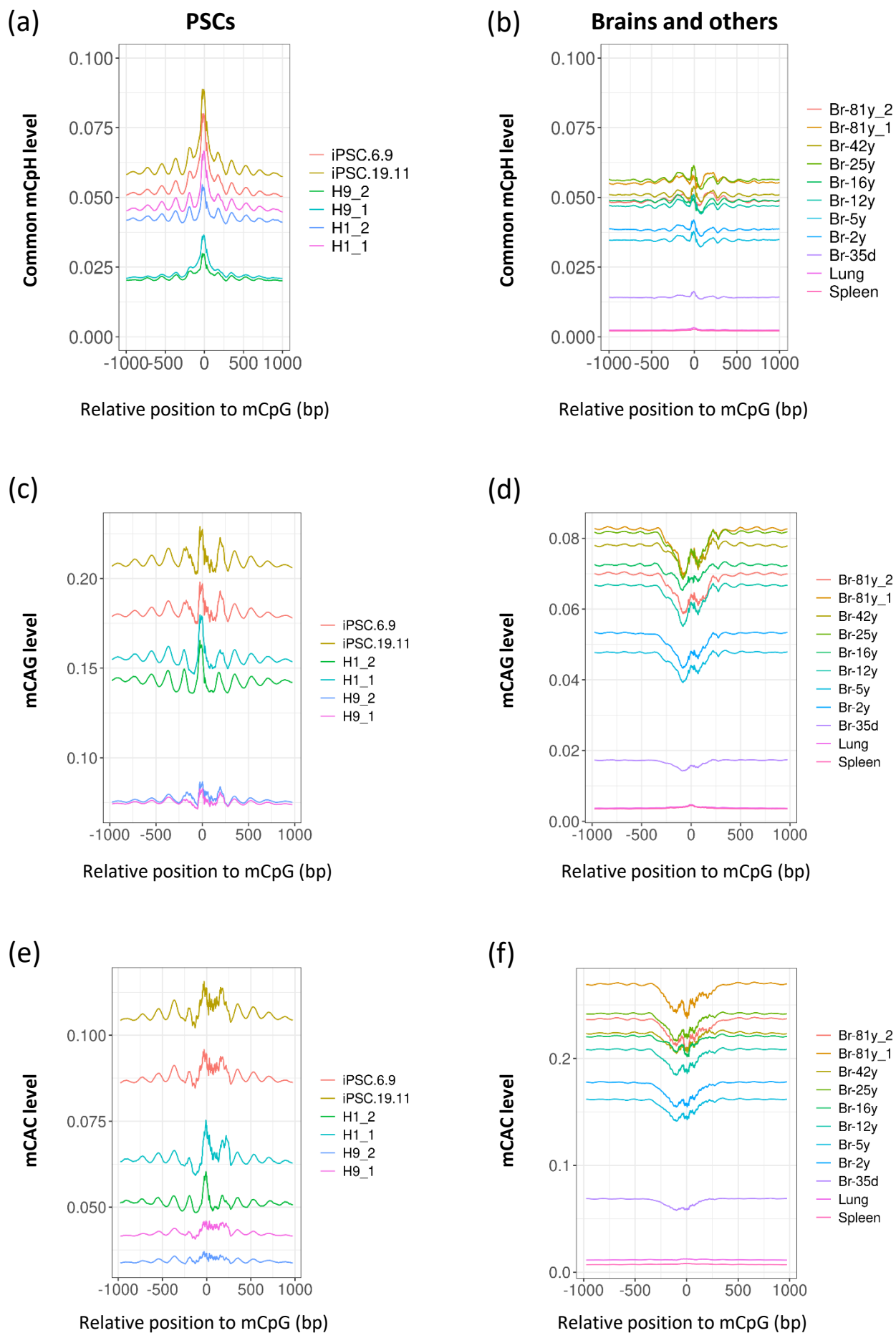

Figure S2

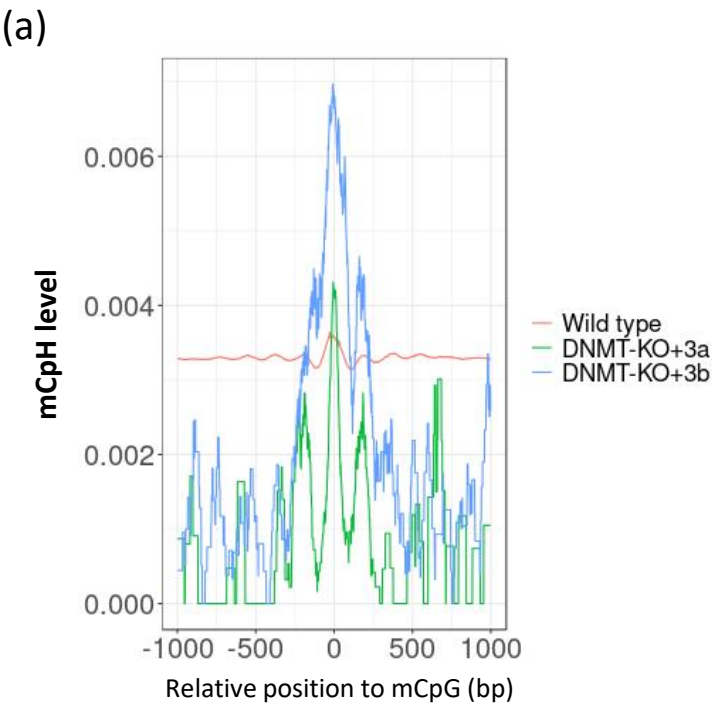

(b)

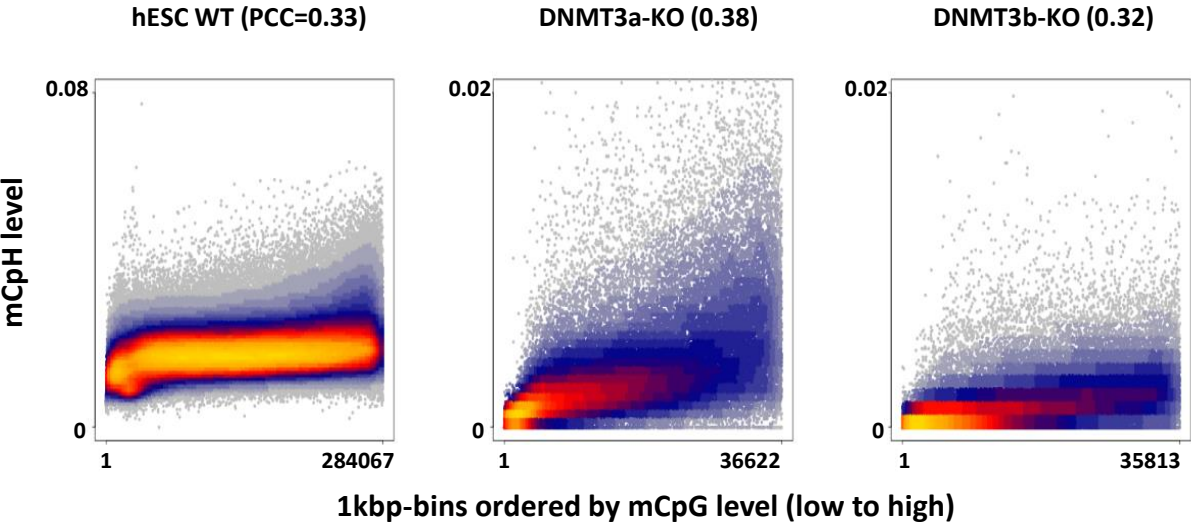

Figure S3

(a)

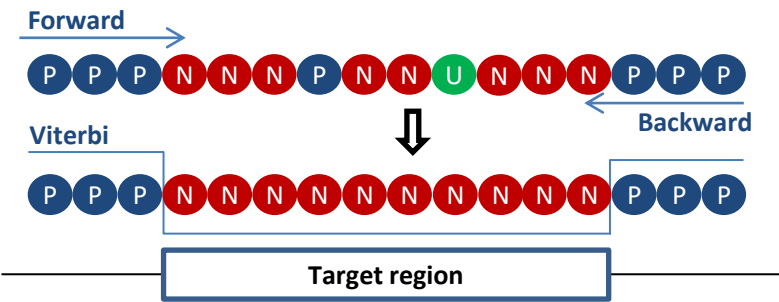

(b)

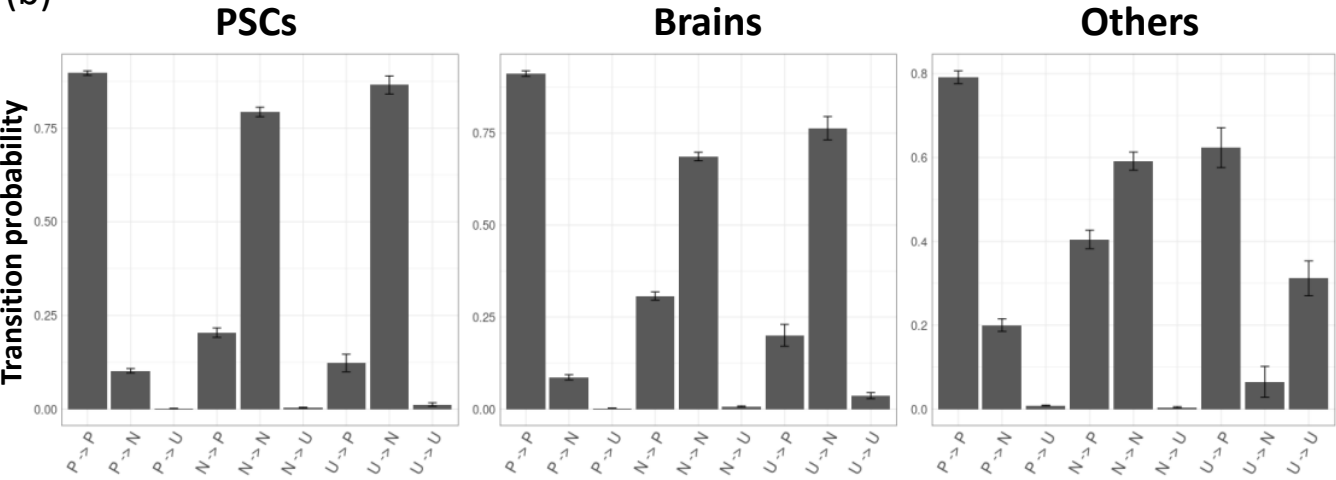

(c)

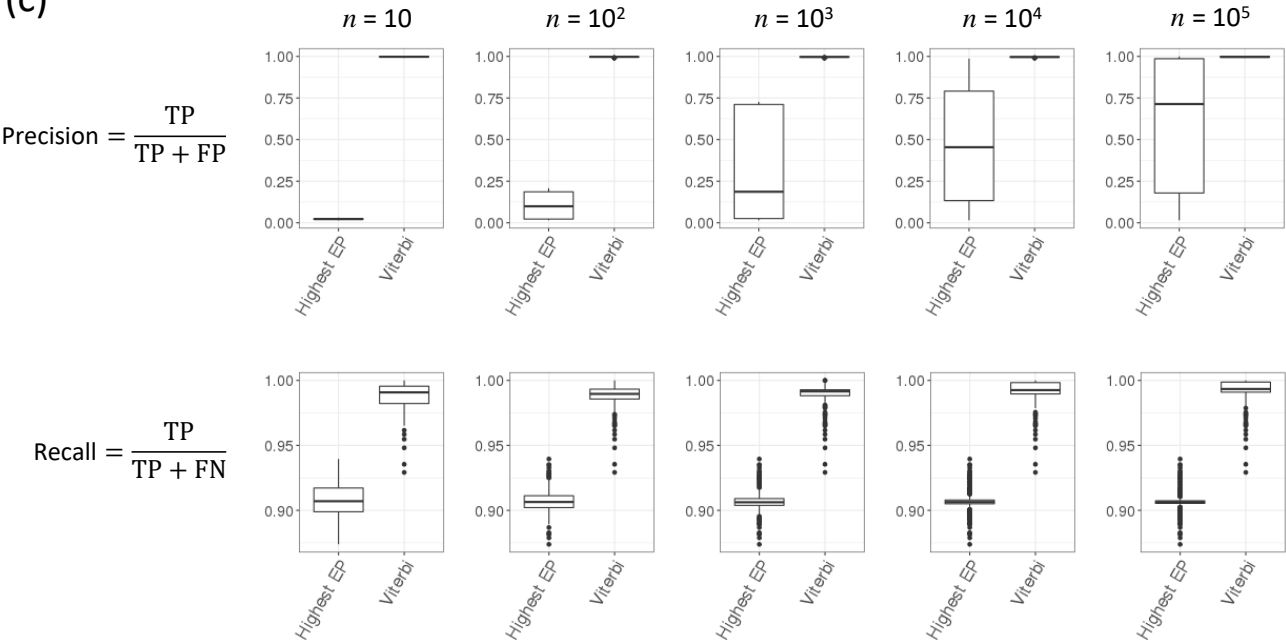

Figure S4

(a)

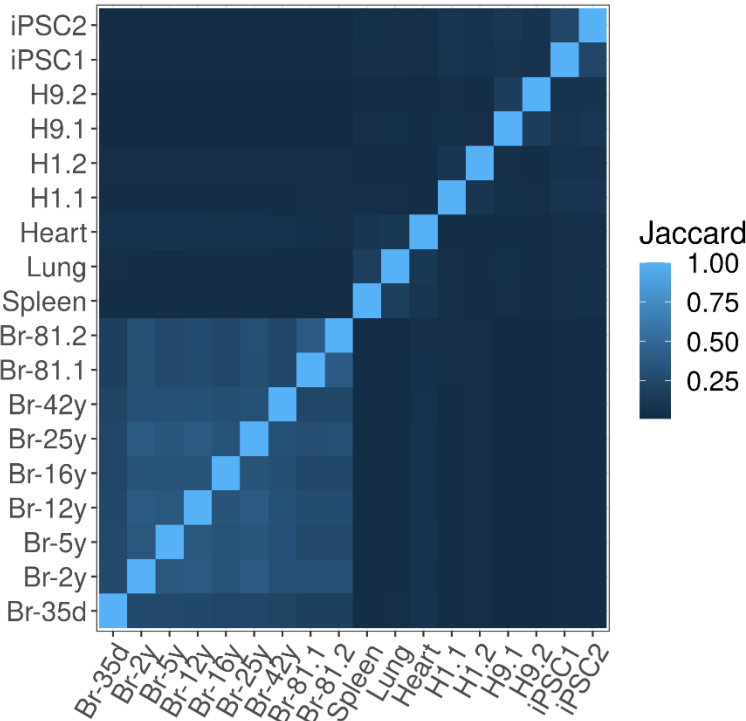

(b)

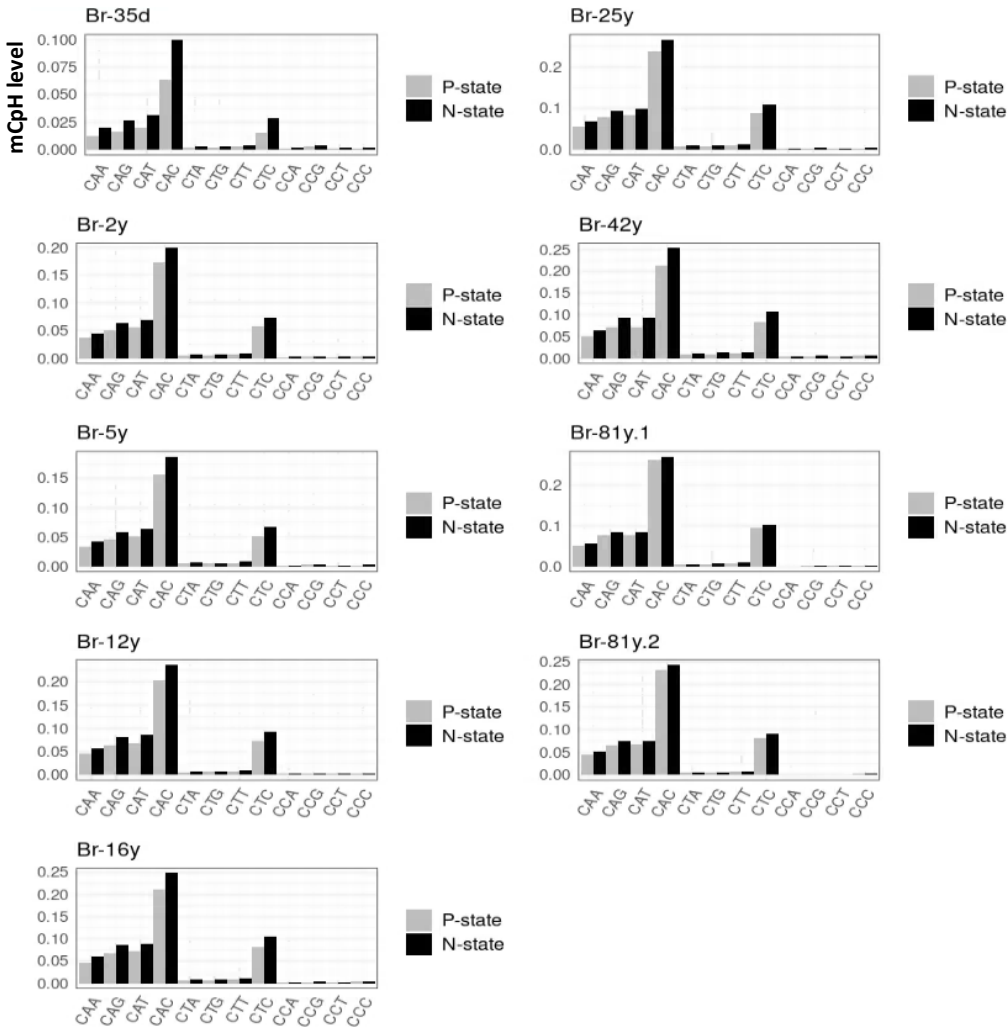

Figure S5

(a)

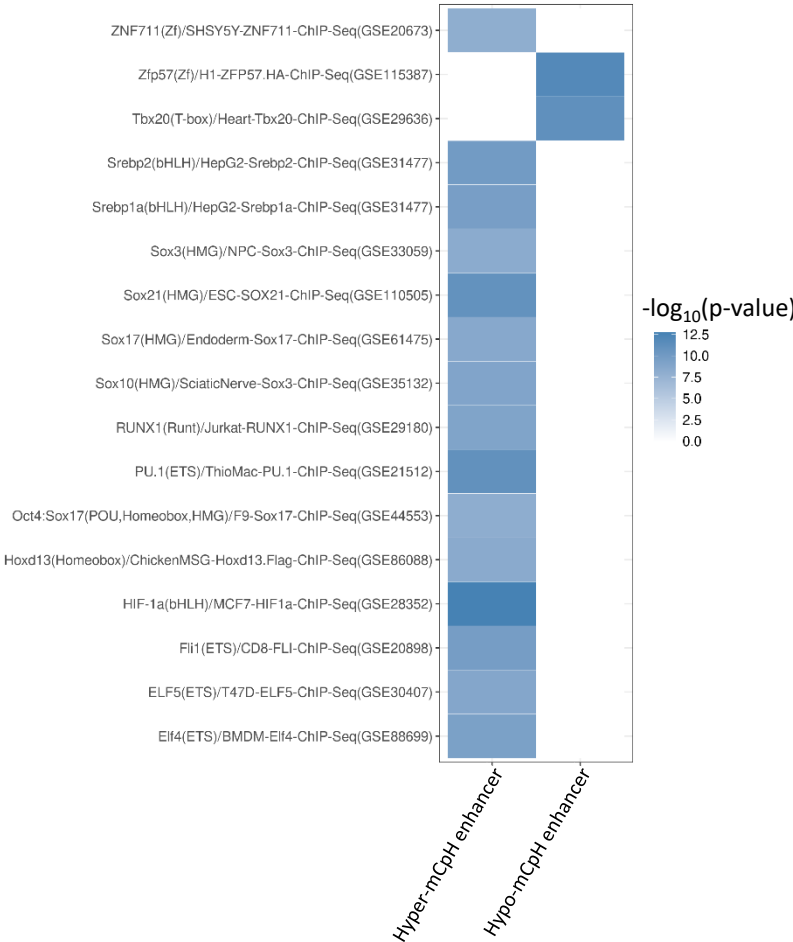

(b)

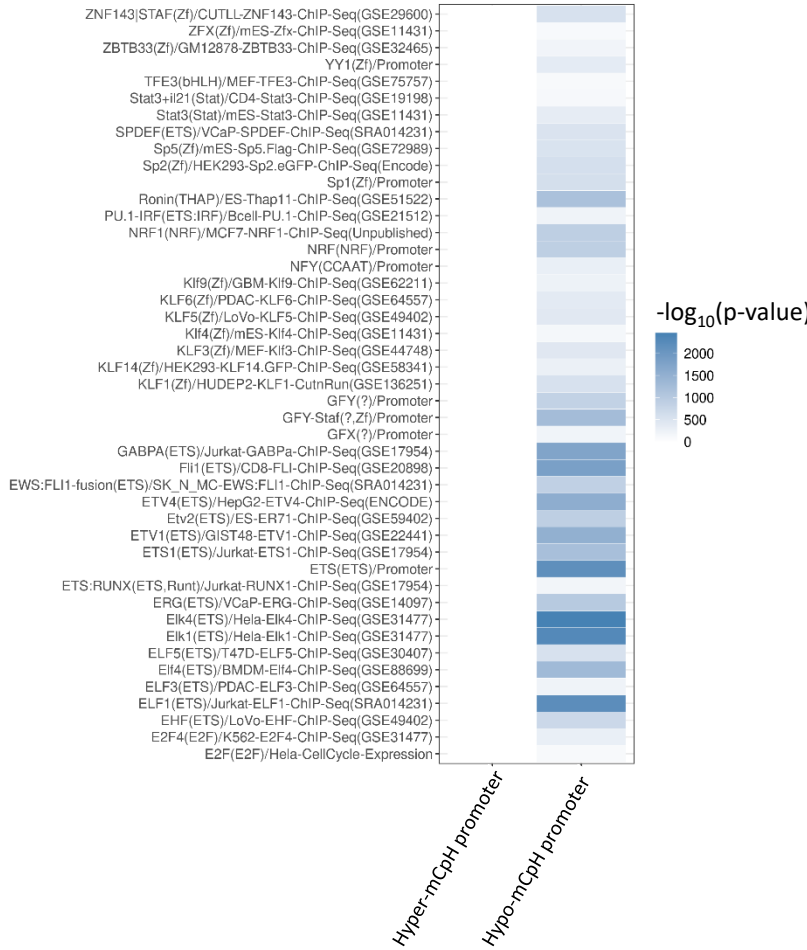

Supplement: dsaa020_Supplementary_Data [file dsaa020_supplementary_data.zip › Supplementary_Figures.pdf]
